# Impact of a Reproductive Health Survivorship Care Plan on Fertility, Pregnancy Concerns, and Accessing Reproductive Healthcare Among Young Nulliparous Breast Cancer Survivors

**DOI:** 10.1002/cnr2.70433

**Published:** 2026-01-08

**Authors:** Ksenya Shliakhtsitsava, Shaylyn S. Stark, Tanushree Prasad, Sarah Boles, Diana Chingos, Jennifer Ehren, Jessica R. Gorman, Sally A. D. Romero, Jun J. Mao, Paul Nathan, Saro H. Armenian, H. Irene Su

**Affiliations:** ^1^ Deparment of Pediatrics, Division of Pediatric Hematolgoy and Oncology University of Texas Southwestern Medical Center Dallas Texas USA; ^2^ Harold C. Simmons Comprehensive Cancer Center University of Texas Southwestern Medical Center Dallas Texas USA; ^3^ Department of Critical Care Palomar Health Escondido California USA; ^4^ School of Nursing, California State University San Marcos San Marcos California USA; ^5^ Division of Pediatrics, Department of Neonatal Intensive Care Providence Regional Medical Center Everett Washington USA; ^6^ Department of Medicine and Moores Cancer Center University of California San Diego California USA; ^7^ Young Survival Coalition New York New York USA; ^8^ Department of Chemical and Biomolecular Engineering University of Notre Dame Notre Dame Indiana USA; ^9^ College of Health School of Nutrition and Public Health Health Promotion and Health Behavior Program Oregon State University Corvallis Oregon USA; ^10^ Department of Obstetrics, Gynecology and Reproductive Science and Moores Cancer Center University of California San Diego California USA; ^11^ Integrative Medicine Service, Memorial Sloan Kettering Cancer Center New York New York USA; ^12^ Division of Hematology/Oncology The Hospital for Sick Children Toronto Canada; ^13^ Department of Pediatrics City of Hope Comprehensive Cancer Center Palo Alto California USA; ^14^ Department of Population Sciences City of Hope Comprehensive Cancer Center Palo Alto California USA

**Keywords:** breast cancer, fertility concerns, pregnancy concerns, randomized controlled trial, reproductive health, survivorship care plan

## Abstract

**Background:**

Young patients with breast cancer frequently receive chemotherapy and/or endocrine therapy that adversely affect ovarian function, leading to fertility, pregnancy, and other reproductive health concerns. Despite available evidence‐based management strategies, dissemination to survivors and healthcare providers remains limited, resulting in substantial unmet informational and care needs. Web‐based survivorship care plans may offer an effective approach to address these gaps.

**Aims:**

To evaluate the effect of a web‐based Reproductive Health Survivorship Care Plan (SCP‐R) on reproductive concerns and reproductive healthcare access among nulliparous young breast cancer survivors (YBCS).

**Methods and Results:**

This is a secondary analysis of a 24‐week randomized controlled trial on the effectiveness of a web‐based SCP‐R addressing unmet informational and clinical management needs for breast cancer survivors aged 18–50 years. The current analysis is restricted to nulliparous participants ages 18–40. The primary outcomes are improvement in fertility and pregnancy health concerns, as measured by the Reproductive Concerns After Cancer (RCAC) scale. The secondary outcome is fertility specialist access measured by referral, consultation, or treatment by a fertility specialist. Among 182 study participants from the parent trial, 47 met the inclusion criteria for the current study (17 in the intervention and 30 in the attention control). Mean age at diagnosis was 30.7 (SD = 3.5) years, and mean age at study participation was 34.0 (SD = 3.8) years. Fertility potential and pregnancy concerns improved in 35.3% of participants randomized to the intervention arm compared to 10.0% in the control arm (RR = 3.5, 95% CI = 1.01–12.34, *p* = 0.05). Intervention arm participants were significantly more likely to receive a fertility specialist referral, schedule a fertility consult, or undergo fertility treatment (37.5% in the intervention arm vs. 6.7% in the control arm; RR = 5.6, 95% CI = 1.28–24.73, *p* = 0.02).

**Conclusions:**

The web‐based SCP‐R intervention led to improvements in fertility potential and pregnancy concerns over time and resulted in more YBCS accessing fertility specialists, highlighting the importance of age‐ and parity‐specific survivorship care interventions to address reproductive health concerns.

## 1 Introduction

There are over 4 million breast cancer survivors in the United States [1]. At diagnosis, 7% of new cases occur in women aged 50 and younger [1]. To be cured, most young breast cancer survivors (YBCS) receive chemotherapy and/or endocrine therapy, treatments that can have a significant impact on reproductive health. Chemotherapy and endocrine therapy may damage ovarian function, resulting in significant reproductive health problems [2, 3]. Hot flashes, sexual dysfunction, limited contraception choices, and infertility may negatively impact survivors' quality of life [4, 5, 6, 7, 8]. Infertility is a distressing consequence of breast cancer treatment among YBCS who desire to have biological children in the future [9, 10]. Evidence‐based clinical strategies geared toward management of reproductive late effects have been developed, but dissemination is limited among YBCS and their healthcare providers [8, 11, 12, 13, 14], leading to significant unmet informational needs among YBCS [15, 16].

The web‐based Survivorship Care Plan on Reproductive Health (SCP‐R) intervention was created to address unmet informational and clinical management needs for YBCS regarding reproductive health‐related problems, including hot flashes, fertility‐related concerns, contraception, and sexual health. Research results, professional society guidelines, and clinical expertise on these issues were combined into management strategies for YBCS and their healthcare providers and delivered via web‐based SCP‐R intervention accessible to both YBCS and their healthcare providers [17, 18]. The parent trial, a randomized controlled trial of 182 YBCS ages 18–50 at the time of enrollment, showed that the SCP‐R intervention was associated with an improvement of at least one of the four targeted issues, that is, hot flashes, fertility‐related concerns, contraception, and sexual health, by 24 weeks [19].

Since fertility potential and pregnancy health‐related concerns tend to be more prevalent in younger cancer survivors and those without children, the objective of the current secondary analysis was to estimate the SCP‐R intervention effect on fertility and pregnancy‐related concerns and fertility care access in nulliparous breast cancer survivors younger than 40 years of age. This younger, childless cohort faces unique reproductive decision‐making challenges not captured in the parent trial results. Therefore, this current study builds on the prior parent trial by isolating the reproductive‐health component of the SCP‐R and identifying its impact on care‐seeking behavior and concerns within a high‐risk, understudied population. We hypothesized that the intervention would have a greater impact on reproductive concerns and fertility specialist access compared to usual care.

## 2 Materials and Methods

We conducted a secondary analysis of a web‐based educational intervention (SCP‐R) on reproductive concerns and fertility care access among nulliparous YBCS younger than 40 years of age. The parent study was a randomized controlled trial on reproductive health issues among YBCS, where study staff, YBCS, and healthcare provider participants were blinded to treatment allocation [17, 19]. Female YBCS were recruited through diverse sources, including cancer advocacy organizations, healthcare providers, patient advocate referrals, Research Match, and previous observational studies conducted by the investigators. The eligibility criteria for the parent trial included current age of 18–50 years, age at diagnosis of 18–45 years, Stage 0–III breast cancer, completion of primary cancer treatment, and one or more of the four women's health issues (fertility or pregnancy concerns, estrogen deprivation symptoms, and/or inadequate contraception). Survivors with ongoing pregnancies were ineligible for the parent trial. After completing an enrollment questionnaire, YBCS participants were randomized in a 1:1 ratio to the intervention and waitlist control arms. All participants were followed longitudinally for 24 weeks and completed questionnaires at 12‐week and 24‐week follow‐ups. The UC San Diego Institutional Review Board approved all study procedures, and the trial was registered at clinicaltrials.gov (NCT02667626).

The SCP‐R intervention included four sections on fertility‐related concerns: (1) a survivorship care plan (SCP) presented in a question‐and‐answer format; (2) a systematic review results summary with online references to primary research articles; (3) a summary of clinical guidelines with online references; and (4) a list of curated additional online resources (Appendix S1). The SCP‐R intervention highlighted actionable steps that would prompt participants to schedule appointments with healthcare providers to address concerns and reproductive health needs. Participants in the intervention arm were given access to the web‐based SCP‐R materials and received biweekly text message reminders focused on reproductive health. Participants in the attention control arm were given access to a list of curated online resources and received text messages on study adherence.

The online enrollment questionnaire focused on demographics, cancer and treatment characteristics, reproductive health and health behaviors, fertility and pregnancy concerns, hot flashes, vaginal symptoms, and contraception. Questionnaires administered at 12 and 24 weeks focused on reproductive health and health behaviors, reproductive concerns, hot flashes, vaginal symptoms, and contraception. Fertility‐related concerns were measured using the Reproductive Concerns After Cancer (RCAC) scale, an 18‐item multidimensional scale composed of individual subscales (fertility potential, pregnancy, child's health, partner disclosure of fertility status, personal health, and acceptance of infertility), measuring reproductive health concerns of young adult female cancer survivors [20]. Each subscale consists of three questions, with response options ranging from 1 (*strongly disagree*) to 3 (*neither agree nor disagree*) to 5 (*strongly agree*). A mean score of 3 or greater on a 5‐point Likert scale of RCAC is used to categorize individuals as having moderate to severe reproductive concerns [21]. In this current study, we focused on fertility potential and pregnancy RCAC subscales, since the SCP‐R intervention content addressed these specific areas (Table S1) [21].

### 2.1 Statistical Analysis

We restricted the analysis to nulliparous YBCS aged 18–40 years. Participants with a history of hysterectomy and/or bilateral oophorectomy were excluded from this analysis (*n* = 3).

Descriptive statistics were calculated as frequencies and percentages or means and standard deviations (SDs), as applicable. The primary outcome of interest was relative improvement in the mean fertility potential or pregnancy concerns subscale score to ≤ 3 by 24 weeks among participants who had moderate/severe concerns (> 3). Scores for the fertility potential and pregnancy subscales were calculated by averaging responses (range: 1–5), with scores > 3 indicating moderate to severe concerns [21]. The secondary outcomes were fertility specialist referral, consultation, and/or fertility treatment by 24 weeks. Bivariate analysis was performed using Fisher's exact test or chi‐square test of proportions to compare outcomes of interest between the intervention and waitlist control arms. Relative risk was calculated for the primary and secondary outcomes. Statistical significance was set at *p* < 0.05. All analyses were conducted using the SAS statistical software.

## 3 Results

**FIGURE 1:**
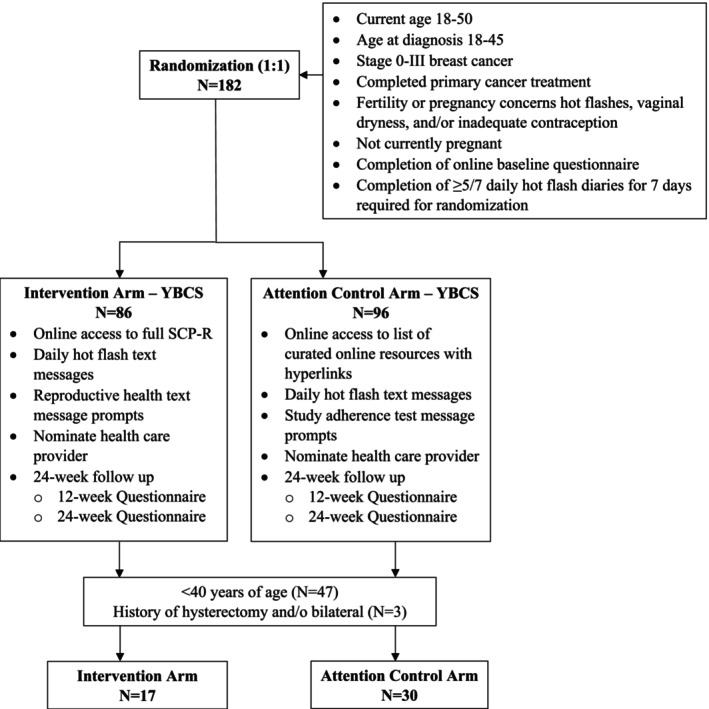
Flow diagram for young breast cancer survivors (YBCS) study participants.

Participants were enrolled between March 2016 and April 2017. Among 182 randomized participants, 47 were eligible for the current analysis (Figure 1). The baseline characteristics and reproductive health issues of the intervention and waitlist control arms are presented in Table 1. The mean age (SD) was 34.2 (±3.8) years. Most of the participants were white (83%), college graduates (93%), and reported being in partnered relationships (66%). Mean age at breast cancer diagnosis (SD) was 30.7 (±3.5) years. Over 50% of the participants were 3 or more years after cancer diagnosis. Breast cancer treatment included surgery (100%), chemotherapy (89%), hormonal therapy (78%), radiation therapy (66%), and biologic therapy (29%). No significant differences were observed between the intervention and waitlist control arms at baseline, except for hormonal treatment exposure (more patients in the control arm were exposed to hormonal therapy [90%] vs. intervention [59%], *p* = 0.02).

**TABLE 1:** Baseline nulliparous YBCS demographic, cancer, and reproductive health‐related characteristics by SCP‐R arm.

Characteristic	Overall	Intervention	Control	*p*
	*N* (%)	*N* (%)	*N* (%)	
	*N* = 47	*N* = 17	*N* = 30	
Age at enrollment (years), mean [SD]	34 ± 3.8	34.3 ± 3.2	33.8 ± 4.1	
25–35	34 (72.3)	13 (76.5)	21 (70.0)	0.76
36–40	13 (27.7)	4 (23.5)	9 (30.0)	0.74
Race				
White	39 (83.0)	16 (94.1)	23 (76.7)	0.29
Black or African American	1 (2.1)	0 (0.0)	1 (3.3)	
Other	7 (14.9)	1 (5.9)	6 (20.0)	
Hispanic ethnicity	2 (4.3)	0 (0.0)	2 (6.7)	0.53
Partnered relationship status	31 (66.0)	10 (58.8)	21 (70.0)	0.44
College graduate	44 (93.6)	17 (100.0)	27 (90.0)	0.29
Income				
< $51 000	12 (26.1)	4 (23.5)	8 (27.6)	1.00
≥ $51 000	34 (73.9)	13 (76.5)	21 (72.4)	
Current smoker	2 (4.3)	1 (5.9)	1 (3.3)	1.00
BMI				
< 18.5	3 (6.4)	1 (5.9)	2 (6.7)	0.15
18.5 to < 25	20 (42.6)	5 (29.4)	15 (50.0)	
25 to < 30	15 (31.9)	9 (52.9)	6 (20.0)	
≥ 30	9 (19.2)	2 (11.8)	7 (23.3)	
Mean age at breast cancer diagnosis [SD]	30.7 ± 3.5	30.5 ± 3.2	30.7 ± 3.8	0.92
Age at diagnosis				
18–24	3 (6.4)	1 (5.9)	2 (6.7)	1.00
25–35	40 (85.1)	15 (88.2)	25 (83.3)	
36–45	4 (8.5)	1 (5.9)	3 (10.0)	
Years since diagnosis				
< 1	7 (14.9)	2 (11.8)	5 (16.7)	0.30
1–2	16 (34.0)	7 (41.2)	9 (30.0)	
3–5	15 (31.9)	3 (17.7)	12 (40.0)	
> 5	9 (19.2)	5 (29.4)	4 (13.3)	
Cancer treatment				
Surgery	47 (100.0)	17 (100.0)	30 (100.0)	1.0
Radiation	31 (66.0)	10 (58.8)	21 (70.0)	0.44
Chemotherapy	42 (89.4)	16 (94.1)	26 (86.7)	0.64
Biologic therapy	14 (29.8)	7 (41.2)	7 (23.3)	0.19
Hormonal treatment	37 (78.7)	10 (58.8)	27 (90.0)	**0.02**
Current GnRH agonist	9 (19.2)	3 (17.7)	6 (20.0)	1.00
Cancer stage				
1	14 (30.4)	3 (18.7)	11 (36.7)	0.15
2	22 (47.8)	11 (68.8)	11 (36.7)	
3	10 (21.7)	2 (12.5)	8 (26.6)	
Comorbidities				
Hypertension	3 (6.4)	1 (5.9)	2 (6.7)	1.00
Diabetes	0 (0.0)	0 (0.0)	0 (0.0)	—
Mental illness	14 (29.8)	3 (17.7)	11 (36.7)	0.17
Osteopenia or osteoporosis	4 (8.5)	2 (11.8)	2 (6.7)	0.61
Menstruation frequency over the past year				
0	14 (29.8)	5 (29.4)	9 (30.0)	0.39
1–3	11 (23.4)	4 (23.5)	7 (23.3)	
4–9	8 (17.0)	1 (5.9)	7 (23.3)	
10–12	14 (29.8)	7 (23.3)	7 (23.3)	
Desires another baby in the future				
No or probably no	9 (19.1)	4 (23.5)	5 (16.7)	0.71
Yes or probably yes	38 (80.9)	13 (76.5)	25 (83.3)	
When will start trying to have a child?				
Not planning	9 (20.0)	3 (17.7)	6 (21.4)	0.92
Attempting now	4 (8.9)	2 (11.8)	2 (7.1)	
< 2 years	17 (37.8)	7 (41.2)	10 (35.7)	
> 2 years	15 (33.3)	5 (29.4)	10 (35.7)	
Trying to become pregnant now				
Yes	4 (8.5)	2 (11.8)	2 (6.7)	0.89
No	32 (68.1)	11 (64.7)	21 (70.0)	
Neither trying nor avoiding	11 (23.4)	4 (23.5)	7 (23.3)	
Healthcare provider talked with me about trying to become pregnant				
Never	7 (14.9)	4 (23.5)	3 (10.0)	0.24
At least once	40 (85.1)	13 (76.5)	27 (90.0)	
Healthcare provider advice on pregnancy				
Avoid	4 (10.0)	0 (0.0)	4 (14.8)	0.35
Attempt now	2 (5.0)	0 (0.0)	2 (7.4)	
Attempt later	30 (75.0)	11 (84.6)	19 (70.4)	
Conflicting advice	4 (10.0)	2 (15.4)	2 (7.4)	
Healthcare provider advice on timing				
No rec	4 (10.0)	1 (7.7)	3 (11.1)	0.71
< 1 year to < 2 years	6 (15.0)	3 (23.1)	3 (11.1)	
2–4 years	9 (22.5)	2 (15.4)	7 (25.9)	
5–10 years	19 (47.5)	7 (53.9)	12 (44.4)	
> 10 years	2 (5.0)	0 (0.0)	2 (7.4)	
Infertility before cancer	1 (2.1)	0 (0.0)	1 (3.3)	1.00
Fertility specialist				
No	14 (29.8)	6 (35.3)	8 (26.7)	0.53
Before cancer	3 (6.4)	1 (5.9)	2 (6.7)	1.00
After cancer before treatment	28 (59.6)	9 (52.9)	19 (63.3)	0.49
During treatment	3 (6.4)	0 (0.0)	3 (10.0)	0.29
After completing treatment	6 (12.8)	2 (11.8)	4 (13.3)	1.00
Fertility concerns subscale				
Mean [SD]	3.9 ± 1.0	4.1 ± 0.7	3.8 ± 1.1	
Mild (≤ 3)	13 (27.7)	2 (11.8)	11 (36.7)	0.58
Moderate/severe (> 3)	34 (72.3)	15 (88.2)	19 (63.3)	0.09
Pregnancy concerns subscale				
Mean [SD]	3.2 ± 0.9	3.3 ± 0.6	3.2 ± 1.0	
Mild (≤ 3)	24 (51.1)	7 (41.2)	17 (56.7)	0.52
Moderate/severe (> 3)	23 (48.9)	10 (58.8)	13 (43.3)	0.31
Personal health subscale				
Mean [SD]	3.3 ± 0.9	3.2 ± 0.9	3.3 ± 0.9	
Mild (≤ 3)	18 (38.3)	7 (41.2)	11 (36.7)	0.54
Moderate/severe (> 3)	29 (61.7)	10 (58.8)	19 (63.3)	0.76
Child health subscale				
Mean [SD]	3.8 ± 0.8	3.8 ± 0.9	3.7 ± 0.8	
Mild (≤ 3)	12 (25.5)	5 (29.4)	7 (23.3)	0.84
Moderate/severe (> 3)	35 (74.5)	12 (70.6)	23 (76.7)	0.73

Sixty‐three percent of the participants desired to have a baby in the future, and an additional 17% reported, while feeling unsure, that they would probably want a baby in the future. Seventy‐two percent of the participants visited a fertility specialist at the time of diagnosis. At baseline, 72% of the participants reported moderate to high fertility potential concerns, 48% reported elevated pregnancy‐related concerns, 61% reported elevated personal health concerns, and 74% had elevated child health concerns. The baseline characteristics were similar between the two arms.

At trial completion, we observed improvement in fertility potential or pregnancy‐related concerns (35.3% improvement in the intervention arm vs. 10.0% improvement in the attention control arm; RR = 3.5, 95% CI = 1.01–12.34, *p* = 0.05) (Table 2). Additionally, intervention arm participants were more likely to have received a fertility specialist referral, scheduled a fertility consultation appointment, or undergone fertility treatment (37.5% in the intervention arm vs. 6.7% in the attention control arm; RR = 5.6, 95% CI = 1.28–24.73, *p* = 0.02). Six individuals in the intervention arm saw a fertility specialist (five individuals received a referral before the visit, and one person scheduled an appointment without a referral); three of those six underwent fertility treatment. In contrast, two individuals in the control arm saw a fertility specialist (one person received a referral and one scheduled an appointment without being referred); one of those two underwent fertility treatment. More participants in the control arm planned or talked with their healthcare provider about managing targeted issues than did those in the intervention arm (*p* = 0.02). We did not observe a significant change in the desire or time horizon to have a baby or personal and child health concerns.

**TABLE 2:** Nulliparous YBCS reproductive health‐related characteristics—change from baseline to 24 weeks by SCP‐R arm and end of study assessment of the intervention result.

Characteristic	Overall	Intervention	Control	*p*
	*N* (%)	*N* (%)	*N* (%)	
	*N* = 47	*N* = 17	*N* = 30	
Change in desire to have a baby				
No change	39 (84.8)	16 (100.0)	23 (76.7)	0.10
Yes to no	6 (13.0)	0 (0.0)	6 (20.0)	
No to yes	1 (2.2)	0 (0.0)	1 (3.3)	
Improvement in fertility or pregnancy concerns at 24 weeks (changed from > 3 to ≤ 3)	9 (19.2)	6 (35.3)	3 (10.0)	**0.05**
Improvement in personal health or child health concerns at 24 weeks (changed from > 3 to ≤ 3)	12 (25.5)	4 (23.5)	8 (26.7)	1.00
Fertility specialist referral, visit, or treatment				
No referral, visit, or treatment	38 (82.6)	10 (62.5)	28 (93.3)	**0.02**
Received referral/appointment/treatment	8 (17.4)	6 (37.5)	2 (6.7)	
Talked with a healthcare provider about managing women's health issues?				
No, thinking about it	4 (8.7)	0 (0.0)	4 (13.3)	**0.02**
No, but plan	6 (13.0)	5 (31.3)	1 (3.3)	
Yes	36 (78.3)	11 (68.8)	25 (83.3)	

## 4 Discussion

A web‐based educational intervention focused on reproductive health was shown to be effective in improving fertility potential or pregnancy‐related concerns among nulliparous YBCS aged 18–40 years and prompted YBCS to seek help from a fertility specialist or other healthcare providers to address their reproductive health concerns. These results support a low‐cost evidence‐based intervention strategy geared toward addressing young cancer survivors' reproductive health needs.

Since breast cancer treatment and its duration can shorten the reproductive window and delay the ability to attain family‐building goals, breast cancer may be even more distressing for reproductive‐aged young females than for older individuals [22, 23, 24]. In our nulliparous YBCS cohort, most participants wanted to have children in the future. Previous studies have shown that young adult female cancer survivors have increased reproductive concerns and unmet informational needs, resulting in a poorer quality of life [4]. Moreover, an increased level of reproductive concerns was associated with greater odds of experiencing moderate‐to‐severe depression among 200 young female cancer survivors [25]. Currently, effective methods to alleviate these reproductive health concerns and disseminate evidence‐based information to YBCS are lacking. The SCP‐R parent trial was the first to address these concerns via a web‐based educational intervention that specifically focused on reproductive health issues [19].

In this current secondary analysis, we observed a greater than threefold improvement in fertility potential and pregnancy‐related concerns among participants randomized to the intervention arm, as measured by the RCAC scale. The RCAC scale is an effective tool for identifying young cancer survivors' concerns related to fertility and parenthood and may be useful for long‐term follow‐up care to assess the reproductive health needs of young women with a history of cancer [20]. Since the original publication on the development of the scale in 2014, multiple studies have applied it [26, 27, 28]. Re‐evaluation of the scale in a sample of 238 female reproductive‐aged cancer survivors (younger than 45 years) suggested that the RCAC may be implemented to assess the six subscale domains and allow for tailoring recommendations and interventions to address specific concerns of female reproductive‐aged cancer survivors [21]. Therefore, in our current study, we focused on the RCAC subscales of fertility potential and pregnancy concerns relevant to the SCP‐R intervention effects at 24 weeks.

Intervention materials were provided in a question‐answer format with the ability to conveniently seek more details from the original resources and guidelines, if desired. It is possible that the information provided in the SCP‐R was sufficient to alleviate some of the fertility potential and pregnancy‐related concerns. This may explain why fewer intervention participants planned to or talked with their healthcare provider about managing targeted issues than those in the control arm. Additionally, the SCP‐R highlighted actionable steps that would prompt participants to schedule appointments with fertility specialists to address reproductive health needs, and more participants in the intervention arm received a fertility specialist referral compared to those in the control arm. Guiding young cancer survivors to fertility specialists is an important goal of oncology and primary care providers that could be facilitated by the SCP‐R or a similar intervention.

SCPs have been proposed as a modality to help survivors navigate survivorship and are currently recommended for all cancer survivors [29]. However, reproductive health‐related survivorship needs are not adequately addressed in current SCPs. A systematic review examining the impact of SCPs showed that although SCPs appear to be feasible and may improve healthcare providers' knowledge of late effects and general survivorship care, there is little evidence that SCPs affect cancer survivors' patient‐reported outcomes [30]. In contrast, our SCP‐R intervention had a positive impact on young cancer survivors' reproductive health concerns and prompted reproductive care‐seeking behaviors. The lack of improvement observed among control group participants, who received only curated online resources with hyperlinks, highlights that providing access to information may not be sufficient to address reproductive health concerns. Rather, more specific reproductive health content and self‐management tools with SCPs may be necessary.

Therefore, our SCP‐R intervention could potentially be incorporated into general SCPs and serve as an additional dialogue‐initiating, personalized tool between cancer survivors and their healthcare providers. Our study highlights the need for SCPs and other interventions to not only inform cancer survivors but also prompt actions and support survivors through decisions related to their reproductive health, including seeking referrals to fertility care. The incorporation and promotion of supportive care programs dedicated to young women with breast cancer, such as the one presented in the study of fertility preservation in young women with breast cancer in Mexico, may be valuable strategies [31]. In addition, interventions such as SCP‐R can serve as an example of late effect‐specific SCPs for cancer survivors (e.g., for reproductive health, cardiac health, and cancer predisposition screening post‐therapy).

Our findings complement and extend those from the parent trial [19], which reported improvement across multiple women's health domains in a broader cohort of YBCS aged 18–50. By focusing exclusively on nulliparous YBCS under 40 years, we demonstrate that the SCP‐R intervention yields a greater than 3‐fold improvement in fertility potential and pregnancy‐related concerns and a 5.6‐fold increase in fertility specialist referral or treatment. Thus, this work uniquely delineates the reproductive health impact of the SCP‐R among those most likely to experience fertility distress, supporting the clinical value of age‐ and parity‐specific tailoring of SCPs. Further, recent studies highlight the global relevance of these findings and emphasize the importance of addressing fertility needs among young cancer survivors from diverse populations [10, 31, 32].

This study had several limitations. First, most participants were white, highly educated, and recruited from advocacy organizations, limiting the generalizability of the results. However, the SCP‐R content can be adapted for other populations, most recently for Spanish‐speaking, Hispanic breast cancer survivors [32]. Second, a higher percentage of participants in the control group required hormone therapy than in the intervention group. This indicates higher rates of hormone‐negative, more aggressive breast cancer in the intervention group, which may have made the two groups less comparable. Third, we had a small number of participants who were 40 years old and younger, with no history of live births. Additionally, the short follow‐up time of 24 weeks makes it difficult to assess whether more YBCS would follow up with a fertility specialist and utilize fertility treatments. Finally, in this study, we relied on self‐reported intentions or actions regarding fertility care. Strategies to overcome these limitations should be considered when designing future studies to improve reproductive care in YBCS.

## 5 Conclusions

While the parent trial included all reproductive‐aged YBCS aged 18–50 years, this secondary analysis focused on a high‐risk subgroup of nulliparous YBCS aged 18–40 years. The web‐based SCP‐R intervention improved fertility potential and pregnancy‐related concerns over time and encouraged access to fertility care, highlighting the importance of age‐ and parity‐specific survivorship care interventions. More research is needed to better understand personalized and targeted approaches for optimal fertility care among nulliparous YBCS and dissemination strategies for the SCP‐R intervention.

## Author Contributions

**Ksenya Shliakhtsitsava:** conceptualization (equal), data curation (equal), formal analysis (equal), investigation (equal), methodology (equal), validation (equal), visualization (equal), writing – original draft (lead), writing – review and editing (lead). **Shaylyn S. Stark:** conceptualization (equal), data curation (equal), formal analysis (equal), funding acquisition (supporting), investigation (supporting), methodology (supporting), project administration (equal), resources (supporting), software (supporting), supervision (equal), validation (equal), visualization (equal), writing – original draft (supporting), writing – review and editing (supporting). **Tanushree Prasad:** formal analysis (supporting), methodology (supporting), software (supporting), writing – review and editing (equal). **Sarah Boles:** investigation (supporting), methodology (supporting), resources (supporting), writing – review and editing (supporting). **Diana Chingos:** conceptualization (equal), data curation (supporting), funding acquisition (supporting), investigation (supporting), methodology (supporting), writing – review and editing (supporting). **Jennifer Ehren:** conceptualization (equal), funding acquisition (supporting), investigation (supporting), methodology (supporting), supervision (supporting), writing – review and editing (supporting). **Jessica R. Gorman:** conceptualization (equal), data curation (equal), formal analysis (supporting), funding acquisition (supporting), investigation (supporting), methodology (equal), project administration (supporting), resources (supporting), writing – review and editing (supporting). **Sally A. D. Romero:** conceptualization (equal), data curation (equal), formal analysis (equal), funding acquisition (supporting), investigation (supporting), methodology (equal), project administration (lead), resources (supporting), software (supporting), supervision (lead), validation (equal), visualization (equal), writing – original draft (supporting), writing – review and editing (supporting). **Jun J. Mao:** conceptualization (equal), funding acquisition (equal), investigation (equal), methodology (equal), supervision (equal), visualization (equal), writing – review and editing (equal). **Paul Nathan:** resources (supporting), supervision (supporting), validation (supporting), visualization (supporting), writing – review and editing (supporting). **Saro H. Armenian:** conceptualization (supporting), methodology (supporting), supervision (supporting), validation (equal), visualization (equal), writing – review and editing (equal). **H. Irene Su:** conceptualization (lead), data curation (lead), formal analysis (lead), funding acquisition (lead), investigation (lead), methodology (lead), project administration (equal), resources (lead), software (lead), supervision (lead), validation (equal), writing – original draft (equal), writing – review and editing (equal).

## Funding

This work was supported by the California Breast Cancer Research Program Translational Award 20OB‐0144 (all authors) and the P30‐CA008748 (J.J.M.). The funders had no involvement in the study design, data collection, analysis, and interpretation of data, writing of the manuscript, or decision to submit the article for publication. Additionally, Ksenya Shliakhtsitsava is a Dedman Family Scholar in Clinical Care.

## Ethics Statement

This study was conducted in accordance with the principles of the Declaration of Helsinki. This study was approved by the Ethics Committee of the University of California, San Diego (IRB Number 140863).

## Consent

Informed consent was obtained from all individual participants included in the study.

## Conflicts of Interest

The authors declare no conflicts of interest.

## Supporting information


**Appendix S1:** Sample of SCP‐R content on fertility‐related concerns for participants randomized to the intervention arm. Similar materials were provided for hot flashes, contraception, sexual health, and cancer genetic risk. Participants randomized to the attention control arm were only able to access the list of curated web‐based resources with hyperlinks.
**Table S1:** The Reproductive Concerns After Cancer (RCAC) scale details. Highlighted in green are the subscales included in this analysis.

## Data Availability

The datasets generated and analyzed during the current study are not publicly available, but are available from the corresponding author upon reasonable request.

## References

[1] N. S. Wagle , L. Nogueira , T. P. Devasia , et al., “Cancer Treatment and Survivorship Statistics, 2025,” CA: A Cancer Journal for Clinicians 75 (2025): 308–340. 40445120 10.3322/caac.70011 PMC12223361

[2] H. I. Su , M. D. Sammel , J. Green , et al., “Antimullerian Hormone and Inhibin B Are Hormone Measures of Ovarian Function in Late Reproductive‐Aged Breast Cancer Survivors,” Cancer 116 (2010): 592–599. 19918920 10.1002/cncr.24746 PMC2815049

[3] S. Yildiz , G. Bildik , C. Benlioglu , et al., “Breast Cancer Treatment and Ovarian Function,” Reproductive Biomedicine Online 46 (2023): 313–331. 36400663 10.1016/j.rbmo.2022.09.014

[4] J. R. Gorman , S. Bailey , J. P. Pierce , and H. I. Su , “How Do You Feel About Fertility and Parenthood? The Voices of Young Female Cancer Survivors,” Journal of Cancer Survivorship 6 (2012): 200–209. 22179785 10.1007/s11764-011-0211-9 PMC3667153

[5] Y. C. Chang , S. R. Chang , and S. C. Chiu , “Sexual Problems of Patients With Breast Cancer After Treatment: A Systematic Review,” Cancer Nursing 42 (2019): 418–425. 29621025 10.1097/NCC.0000000000000592

[6] H. Y. Chang , A. C. Jotwani , Y. H. Lai , et al., “Hot Flashes in Breast Cancer Survivors: Frequency, Severity and Impact,” Breast 27 (2016): 116–121. 27065357 10.1016/j.breast.2016.02.013 PMC5893329

[7] J. Howard‐Anderson , P. A. Ganz , J. E. Bower , and A. L. Stanton , “Quality of Life, Fertility Concerns, and Behavioral Health Outcomes in Younger Breast Cancer Survivors: A Systematic Review,” Journal of the National Cancer Institute 104 (2012): 386–405. 22271773 10.1093/jnci/djr541

[8] M. Peate , B. Meiser , M. Hickey , and M. Friedlander , “The Fertility‐Related Concerns, Needs and Preferences of Younger Women With Breast Cancer: A Systematic Review,” Breast Cancer Research and Treatment 116 (2009): 215–223. 19390962 10.1007/s10549-009-0401-6

[9] A. L. Carr , S. Roberts , L. N. Bonnell , and E. Kolva , “Existential Distress and Meaning Making Among Female Breast Cancer Patients With Cancer‐Related Fertility Concerns,” Palliative & Supportive Care 21 (2023): 196–204. 36562084 10.1017/S1478951522001675

[10] L. Chen , J. Shen , H. Jiang , et al., “Incidence and Influencing Factors of Fertility Concerns in Breast Cancer in Young Women: A Systematic Review and Meta‐Analysis,” Frontiers in Oncology 13 (2023): 1273529. 38173831 10.3389/fonc.2023.1273529 PMC10763242

[11] D. Buske , A. Sender , D. Richter , E. Brähler , and K. Geue , “Patient‐Physician Communication and Knowledge Regarding Fertility Issues From German Oncologists' Perspective—A Quantitative Survey,” Journal of Cancer Education 31 (2016): 115–122. 25934223 10.1007/s13187-015-0841-0

[12] B. Thewes , B. Meiser , A. Taylor , et al., “Fertility‐ and Menopause‐Related Information Needs of Younger Women With a Diagnosis of Early Breast Cancer,” Journal of Clinical Oncology 23 (2005): 5155–5165. 16051957 10.1200/JCO.2005.07.773

[13] S. K. Nguyen and P. A. Ingledew , “Tangled in the Breast Cancer Web: An Evaluation of the Usage of Web‐Based Information Resources by Breast Cancer Patients,” Journal of Cancer Education 28 (2013): 662–668. 23832398 10.1007/s13187-013-0509-6

[14] E. K. Maloney , T. A. D'Agostino , A. Heerdt , et al., “Sources and Types of Online Information That Breast Cancer Patients Read and Discuss With Their Doctors,” Palliative & Supportive Care 13 (2015): 107–114. 24182945 10.1017/S1478951513000862 PMC4515565

[15] C. Benedict , B. Thom , D. N. Friedman , et al., “Young Adult Female Cancer Survivors' Unmet Information Needs and Reproductive Concerns Contribute to Decisional Conflict Regarding Posttreatment Fertility Preservation,” Cancer 122 (2016): 2101–2109. 27213483 10.1002/cncr.29917 PMC4911318

[16] J. R. Gorman , P. M. Usita , L. Madlensky , and J. P. Pierce , “Young Breast Cancer Survivors: Their Perspectives on Treatment Decisions and Fertility Concerns,” Cancer Nursing 34 (2011): 32–40. 20697269 10.1097/NCC.0b013e3181e4528d PMC2980796

[17] S. S. Stark , L. Natarajan , D. Chingos , et al., “Design of a Randomized Controlled Trial on the Efficacy of a Reproductive Health Survivorship Care Plan in Young Breast Cancer Survivors,” Contemporary Clinical Trials 77 (2019): 27–36. 30553078 10.1016/j.cct.2018.12.002 PMC6754982

[18] J. R. Gorman , A. K. Julian , S. A. Roberts , et al., “Developing a Post‐Treatment Survivorship Care Plan to Help Breast Cancer Survivors Understand Their Fertility,” Support Care Cancer 26 (2018): 589–595. 28913697 10.1007/s00520-017-3871-9 PMC7413066

[19] H. Irene Su , S. Stark , B. Kwan , et al., “Efficacy of a Web‐Based Women's Health Survivorship Care Plan for Young Breast Cancer Survivors: A Randomized Controlled Trial,” Breast Cancer Research and Treatment 176 (2019): 579–589. 31054032 10.1007/s10549-019-05260-6 PMC6626763

[20] J. R. Gorman , H. I. Su , J. P. Pierce , S. C. Roberts , S. A. Dominick , and V. L. Malcarne , “A Multidimensional Scale to Measure the Reproductive Concerns of Young Adult Female Cancer Survivors,” Journal of Cancer Survivorship 8 (2014): 218–228. 24352870 10.1007/s11764-013-0333-3 PMC4016119

[21] J. R. Gorman , T. M. Pan‐Weisz , J. H. Drizin , H. I. Su , and V. L. Malcarne , “Revisiting the Reproductive Concerns After Cancer (RCAC) Scale,” Psycho‐Oncology 28 (2019): 1544–1550. 31128074 10.1002/pon.5130 PMC8428791

[22] R. Peled , D. Carmil , O. Siboni‐Samocha , and I. Shoham‐Vardi , “Breast Cancer, Psychological Distress and Life Events Among Young Women,” BMC Cancer 8 (2008): 245. 18721454 10.1186/1471-2407-8-245 PMC2527614

[23] A. Romeo , M. di Tella , A. Ghiggia , et al., “The Traumatic Experience of Breast Cancer: Which Factors Can Relate to the Post‐Traumatic Outcomes?,” Frontiers in Psychology 10 (2019): 891. 31105621 10.3389/fpsyg.2019.00891 PMC6491882

[24] L. D. Butler , C. Koopman , C. Classen , and D. Spiegel , “Traumatic Stress, Life Events, and Emotional Support in Women With Metastatic Breast Cancer: Cancer‐Related Traumatic Stress Symptoms Associated With Past and Current Stressors,” Health Psychology 18 (1999): 555–560. 10619528 10.1037//0278-6133.18.6.555

[25] J. R. Gorman , H. I. Su , S. C. Roberts , S. A. Dominick , and V. L. Malcarne , “Experiencing Reproductive Concerns as a Female Cancer Survivor Is Associated With Depression,” Cancer 121 (2015): 935–942. 25377593 10.1002/cncr.29133 PMC4352116

[26] P. Anandavadivelan , M. Wiklander , L. E. Eriksson , L. Wettergren , and C. Lampic , “Cultural Adaptation and Psychometric Evaluation of the Swedish Version of the Reproductive Concerns After Cancer (RCAC) Scale,” Health and Quality of Life Outcomes 18 (2020): 273. 32762775 10.1186/s12955-020-01520-y PMC7412787

[27] T. T. Qiao , W. Zheng , W. Xing , et al., “Psychometric Properties of the Chinese Version of the Reproductive Concerns After Cancer Scale (RCAC) for Young Female Cancer Survivors,” Support Care Cancer 25 (2017): 1263–1270. 27921223 10.1007/s00520-016-3519-1

[28] A. Bártolo , I. M. Santos , E. Valério , et al., “The European Portuguese Version of the Reproductive Concerns After Cancer Scale (RCACS): A Psychometric Validation for Young Adult Female Cancer Survivors,” European Journal of Oncology Nursing 47 (2020): 101781. 32563843 10.1016/j.ejon.2020.101781

[29] Institute of Medicine . Implementing Cancer Surviovrship Care Planning: Workshop Sumary. (National Academies Press, 2007), 10.17226/11739.

[30] R. E. Hill , C. E. Wakefield , R. J. Cohn , et al., “Survivorship Care Plans in Cancer: A Meta‐Analysis and Systematic Review of Care Plan Outcomes,” Oncologist 25 (2020): e351–e372. 32043786 10.1634/theoncologist.2019-0184 PMC7011634

[31] C. Villarreal‐Garza , F. Mesa‐Chavez , A. Plata de la Mora , et al., “Prospective Study of Fertility Preservation in Young Women With Breast Cancer in Mexico,” Journal of the National Comprehensive Cancer Network 19 (2021): 1–8, 10.6004/jnccn.2020.7692. 34153944

[32] S. A. D. Romero , H. Palomino , S. H. Ahmed , et al., “Intervening on Women's Health for Rural Young Breast Cancer Survivors: A Study Protocol,” Contemporary Clinical Trials 130 (2023): 107215. 37164298 10.1016/j.cct.2023.107215 PMC10723631

