# Supplementary material for: Impact of a Reproductive Health Survivorship Care Plan on Fertility, Pregnancy Concerns, and Accessing Reproductive Healthcare Among Young Nulliparous Breast Cancer Survivors
Source: Cancer Rep (Hoboken). 2026 Jan 8;9(1):e70433. doi: 10.1002/cnr2.70433 (PMC12782588; doi:10.1002/cnr2.70433)
Supplement: Supplementary file 1 — Appendix S1: Sample of SCP‐R content on fertility‐related concerns for participants randomized to the intervention arm. Similar materials were provided for hot flashes, contraception, sexual health, and cancer genetic risk. Participants randomized to the attention control arm were only able to access the list of curated web‐based resources with hyperlinks. Table S1: The Reproductive Concerns After Cancer (RCAC) scale details. Highlighted in green are the subscales included in this analysis. [file CNR2-9-e70433-s001.pdf]

FERTILITY CONCERNS IN WOMEN WITH BREAST CANCER

Welcome to the **Fertility Concerns Section** of the **Women's Health Survivorship Care Plan (SCP)** for young breast cancer survivors.

This section includes:

1. **Fertility Concerns SCP:**

   Review this short version of the Fertility Concerns SCP for take-home points and action steps. The **Fertility Concerns SCP** can be read online or printed as a 3-page pdf document.

2. **What does the evidence show?**

   Find a summary of clinical research studies on fertility concerns in breast cancer survivors.

3. **What do clinical guidelines say?**

   See a summary of professional healthcare society guidelines on fertility issues in breast cancer survivors.

4. **Resources**

   Take a look at this curated list of helpful online resources on fertility concerns in breast cancer survivors.

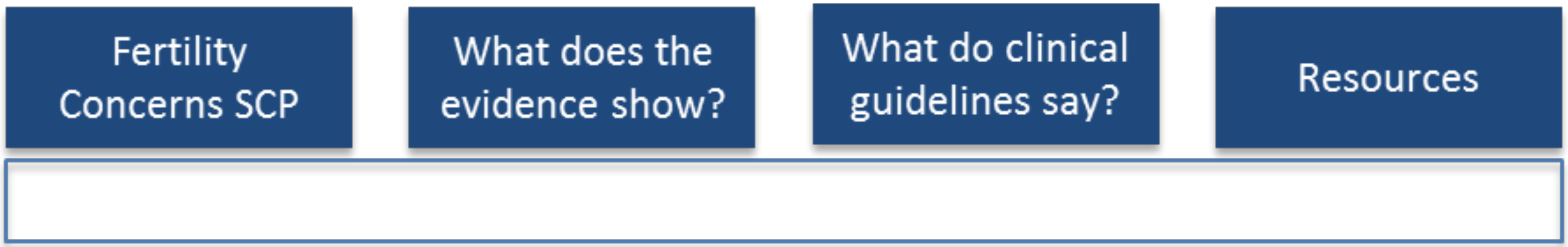

UC San Diego
SCHOOL OF MEDICINE

Fertility Concerns After Breast Cancer Treatment

Is it safe to become pregnant?

- Research studies suggest that pregnancy after breast cancer does not raise the risk of cancer recurrence or affect survival, regardless of estrogen receptor status.
- There is no specific recommendation on how long breast cancer survivors should wait after cancer treatment until they try to become pregnant. That is why women should discuss the timing of pregnancy and their personal health concerns with their healthcare providers.
- Along with life situation, ideal timing depends on completion of cancer treatment, risk of relapse, age, and fertility potential.

What are family building options after finishing breast cancer treatment?

- Using eggs and embryos obtained from fertility preservation **before** cancer treatment to become pregnant.
- **Fertility preservation after cancer treatment** (such as freezing eggs or embryos) is possible for some women. Women should discuss this with their oncology provider and a fertility specialist.
  - The cost of fertility preservation varies widely, but is usually between $3,000 and $15,000, and is often not covered by health insurance.
  - A fertility consultation may be covered. Ask your insurance company.
- **Other options** include getting donor eggs or embryos, using a surrogate mother to carry a pregnancy, adoption, and childfree living.

How can a breast cancer survivor tell if she is fertile (able to have children)?

1) **Attempt pregnancy**

- The best way to predict fertility after breast cancer is to try to become pregnant.

2) **Monitor menstrual bleeding (periods)**

- Having monthly periods is a sign that the ovaries are working, but does not always mean fertility.
- Common types of chemotherapy can damage eggs. This interrupts ovulation and results in temporary or permanent loss of periods.
  - Many women who stop getting their periods will see them return within 6 months of finishing chemotherapy, but it can take longer.
  - Those women who do not stop getting periods or who get them back after chemotherapy still may have damage to their eggs. This could result in infertility (and menopause) at a younger age than expected.
  - Women over the age of 40 have a higher chance that chemotherapy will lead to permanent loss of periods (menopause).
- Medicines called gonadotropin releasing hormone agonists (GnRHa, such as Lupron and Zoladex) can suppress ovulation and temporarily stop periods. Many women will get their periods back within 6 months of stopping GnRHa, but it can take longer.
- Most women taking tamoxifen will continue to ovulate, even though many will have fewer and less regular periods.
- In women with no cancer treatment, menopause happens between age 40 and 60 (average age 51) after there are no periods for 12 months.

Fertility Concerns information comes from review of published research studies, professional society guidelines and clinical expert opinions, current through June, 2015.

3) A fertility specialist can monitor the number and quality of eggs in the ovaries, called ovarian reserve testing.

- Ovarian reserve is the number and quality of eggs that remain in a woman's ovaries.
- Ovarian reserve testing can help measure a woman's fertility potential.
- Common tests include blood tests of follicle stimulating hormone (FSH) and anti-mullerian hormone (AMH), as well as ultrasound tests of antral follicle count (AFC). FSH is best tested with estradiol between menstrual cycle days 3 and 5, while AMH and AFC can be tested at any time.
- As women age and fertility potential declines, FSH levels increase, while AMH levels and AFC decrease to reflect decreasing ovarian reserve.
- Chemotherapy can lower the number and quality of eggs in the ovaries, resulting in lower AMH and AFC and higher FSH. But these levels can fluctuate after treatment.
- Test results provide clues, but cannot accurately predict fertility after breast cancer because there hasn't been enough research on this topic.

Will fertility be affected by using tamoxifen?

- **It is possible to become pregnant while on tamoxifen.** Because tamoxifen can cause fetal harm if given to a pregnant woman, **women should wait at least 2 months after stopping tamoxifen to try and become pregnant**.
- Tamoxifen does not have a permanent effect on fertility or reduce the number or quality of eggs that remain in the ovaries. But fertility will decline with natural aging during the time that women are taking tamoxifen.

Will fertility be affected in women who have a BRCA1 or BRCA2 gene mutation?

- Women who have BRCA1 or BRCA2 gene mutations may start menopause at a slightly earlier age, but most studies show no difference in total number of births, fertility problems, or use of fertility treatment for BRCA carriers compared to non-carriers.
- Because young women who carry BRCA1 or BRCA2 gene mutations have higher risks of developing breast and ovarian cancers, risk-reducing salpingo-oophorectomy (removal of the fallopian tubes and ovaries) to prevent ovarian cancer is typically recommended by age 40 years or when childbearing is complete.
- Embryos from assisted reproduction may be checked for BRCA1 or BRCA 2 mutations through genetic testing before they are implanted.

Will fertility be affected by using a GnRH agonist (for example, Lupron) during chemotherapy?

- It is possible that ovarian suppression (shutting down the ovaries) during chemotherapy using GnRH agonists such as leuprolide (Lupron), goserelin (Zoladex), triptorelin (Trelstar), and buserelin (Suprefact), increases the chance of fertility after cancer, but there is not enough research to know for sure.
- Because these medicines block ovulation, women on continuous GnRH agonists do not have menstrual bleeding and cannot become pregnant.

Key Online Resources

- Society for Assisted Reproductive Technology (ART information and fertility specialists search): www.sart.org
- Livestrong fertility (fertility risks from treatment): www.livestrong.org/we-can-help/fertility-services
- Oncofertility Consortium (fertility preservation information): http://www.myoncofertility.org

Next steps if you want to have children in the future:

1. **Discuss** with oncologist your desire to have children
2. **Get referred** to a fertility specialist
3. **Discuss** family building options with fertility specialist
4. **Choose** the best family building option for you

Glossary

- AFC: Antral follicle count is the number of ovarian follicles from both ovaries measured by vaginal ultrasound. AFC helps to gauge how many eggs remain in the ovaries.
- AMH: Anti-mullerian hormone is produced by nurse cells surrounding eggs in the ovaries. Blood tests for AMH help gauge how many eggs remain in the ovaries.
- BRCA: BRCA1 and BRCA2 are human genes. Genetic mutations, or alterations of these genes, are linked to an increased risk of breast and ovarian cancer.
- Estradiol: A human sex hormone that plays an important role in the female reproductive cycle.
- FSH: Follicle stimulating hormone is produced by the pituitary gland. FSH helps control the menstrual cycle and production of eggs in the ovaries. FSH helps to gauge how many eggs remain in the ovaries.
- Ovarian follicle: Structures in the ovary that enclose human eggs.
- Ovarian reserve: Number and quality of eggs that remain in the ovaries.
- Ovarian suppression: Preventing the ovaries from making estrogen and ovulation. Also sometimes called shutting down the ovaries or putting the ovaries to sleep.
- Progesterone: A hormone produced by the ovaries during release of a mature egg from an ovary (ovulation).
- Salpingo-oophorectomy: Removal of fallopian tubes and ovaries.

What does the evidence show?

In June, 2015, researchers at UC San Diego performed a systematic review of clinical research studies on fertility concerns in breast cancer survivors. Below are the main findings on:

- **Safety of pregnancy after breast cancer**
  - **There are no specific professional healthcare society recommendations on how long breast cancer survivors should wait after cancer treatment until they try to become pregnant.**
  - Young breast cancer survivors should discuss the timing of pregnancy and their personal health concerns with their healthcare providers. Along with life situation, ideal timing depends on completion of cancer treatment, risk of relapse, age, and fertility potential.
  - Research studies suggest that pregnancy after breast cancer does not raise the risk of cancer recurrence or affect survival, regardless of estrogen receptor status.
  - Because chemotherapy or tamoxifen can cause fetal harm if given to a pregnant woman, women should wait <u>at least</u> 2 months after stopping tamoxifen and 3-6 months after stopping chemotherapy to try and become pregnant.
- **Assessing fertility after breast cancer treatment**
  1. **Attempt pregnancy**: The best way to predict fertility after breast cancer is to try to become pregnant.
  2. **Monitoring menstrual bleeding (periods)** can help gauge fertility potential.
     - Having monthly periods is a sign of ovarian function, i.e. the ovaries are working, but does not always mean fertility. For example, a 50 year old woman may have menstrual bleeding, but not be fertile because of the quantity and quality of her eggs.
     - Menstrual bleeding can be affected by chemotherapy, gonadotropin releasing hormone agonists and tamoxifen.
     - **Chemotherapy** can damage eggs. This interrupts ovulation and results in temporary or permanent loss of periods.
       - Many women who stop getting their periods will see them return within 6 months of finishing chemotherapy, but it can take longer.
       - If there is no menstrual bleeding for two years after chemotherapy, then it is likely that it won’t come back. There is a small (less than 10%) chance that menstruation will return.

        - Women over the age of 40 have a higher chance that chemotherapy will lead to permanent loss of periods (menopause).
    - **Gonadotropin releasing hormone agonists** (GnRHa, such as Lupron and Zoladex) do not damage eggs. But this medication will temporarily prevent ovulation and stop periods. Periods usually resume within 3 to 6 months of stopping the treatment.
    - Most women taking **tamoxifen** will continue to ovulate, even though many will have fewer and less regular periods. Tamoxifen does not damage eggs.
    - In women with no cancer treatment, menopause happens between age 40 and 60 (average age 51) after there are no periods for 12 months.

3. **Ovarian reserve testing** can help gauge fertility potential.
    - Ovarian reserve refers to the number and quality of eggs that remain in a woman's ovaries.
    - Common ovarian reserve tests include blood tests of follicle stimulating hormone (FSH) and anti-mullerian hormone (AMH), as well as ultrasound tests of antral follicle count (AFC).
    - If a woman is having menstrual periods, FSH is best tested with estradiol between menstrual cycle days 3 and 5, while AMH and AFC can be tested at any time.
    - As women age and fertility potential declines, FSH levels increase, while AMH levels and AFC decrease to reflect lower ovarian reserve.
    - Ovarian reserve tests can help provide information on a woman's fertility potential. However, for all of these ovarian reserve, there is no laboratory cut off level that definitively predicts fertility.
    - Ovarian reserve test results can also change over time, so one-time results may not reflect the true ovarian reserve. Research is needed to find out when and how often to measure ovarian reserve after the end of cancer treatment.

- **Birth outcomes after breast cancer**
    - Research suggests no increased risk of cancer in children of cancer survivors, except in cases of heritable cancers. For example, BRCA1 and 2 mutation carriers have a 50% chance of passing the mutation on to their children. Children who inherit a BRCA1 or BRCA2 mutation from their parent would be at higher risk of developing cancer.
    - Limited research suggests no increased risk of birth defects and chromosomal abnormalities for children who were conceived after completing breast cancer treatment.

- Some research studies suggest that women previously treated for invasive breast cancer may have slightly more delivery complications, cesarean deliveries, preterm births, and babies born with a low birth weight compared to the general population.
- There were no studies on other health or developmental outcomes in children who were born after their mothers were treated for breast cancer.

How does breast cancer or breast cancer treatments affect fertility?

- There is no evidence that breast cancer itself affects fertility.
- **Chemotherapy** drugs used in breast cancer, such as cyclophosphamide (Cytoxan), cause direct damage to eggs, which can leave fewer or lower quality eggs.
- **Radiation** to the chest and axilla does not affect ovarian reserve. Only abdominal and pelvic radiation affects ovarian reserve because this puts the ovaries and eggs at risk for radiation injury.
- **Gonadotropin releasing hormone (GnRH) agonists,** such as leuprolide (Lupron) or goserlin (Zoladex), are sometimes given to suppress ovarian function during and following chemotherapy. These medicines may preserve fertility and ovarian function in premenopausal females undergoing gonadotoxic chemotherapy.
  - Compared to chemotherapy alone, GnRH agonists given before and during chemotherapy may increase the likelihood of return of menstruation. This finding is seen in multiple but not all randomized clinical trials.
  - Fertility after breast cancer treatment has been reported in one study of premenopausal breast cancer patients (POEMS). Women who received cyclophosphamide-based chemotherapy were randomized to receive monthly goserelin (3.6 mg SQ) during chemotherapy or no goserelin. Among 135 participants, the ovarian failure rate was lower in the women who received the GnRH agonist than the women who did not (8% versus 22%, odds ratio 0.3 [95% confidence interval 0.09 to 0.97]; p=0.04). Pregnancy also occurred in more women who received the GnRH agonist than the women who did not (21% vs. 11%, p=0.03).
- **Antibodies:** There are no research studies on whether ovarian reserve is affected in premenopausal women taking **trastuzumab (Herceptin)** or **Pertuzumab (Perjeta)**, sometimes used to treat patients with HER2-positive breast cancer.
- **Bevacizumab (Avastin):** Premenopausal women with colon cancer who receive bevacizumab may have higher risk of ovarian failure. There are no data reported on women with breast cancer.
- **Tamoxifen** does not have a permanent effect on fertility or reduce the number or quality of eggs that remain in the ovaries, beyond what happens with normal aging.
  - Women should avoid pregnancy while on tamoxifen and for at least two months after stopping.

        - Tamoxifen has estrogenic effects on the developing genital tract in experimental animals and may cause fetal harm if given to a pregnant woman.
        - It is possible to become pregnant while on tamoxifen. Women should consistently use an effective contraception method while on tamoxifen to avoid pregnancy.
    - **Aromatase Inhibitors** such as anastrozole (Arimidex), exemestane (Aromasin), and letrozole (Femara) are a type of endocrine therapy. They do not have a permanent effect on fertility, beyond what happens with normal aging.

- **BRCA1 or BRCA2 gene mutation carriers**
    - There is conflicting evidence about whether BRCA mutation status affects fertility.
        - Most studies show that BRCA mutation carriers have the same total number of births, fertility problems, or use of fertility treatment as non-carriers.
        - There are also conflicting studies on whether BRCA1 carriers have lower ovarian reserve measured by hormones such as AMH compared to non-carriers.
        - Some studies find that BRCA mutation carriers start menopause at a slightly earlier age than non-carriers or that BRCA1 carriers produced fewer eggs than non-carriers while undergoing fertility treatment.
    - Embryos from assisted reproduction may be checked for BRCA1 or BRCA2 mutations through genetic testing before they are implanted.
    - Completing childbearing earlier would allow for earlier removal of the fallopian tubes and ovaries, which reduces cancer risk. This is recommended at age 35- 40 years or when childbearing is complete. Removing both ovaries means a woman cannot use her own eggs to have a child, unless she has her eggs or embryos frozen prior to surgery.

What do clinical guidelines say on fertility after breast cancer treatment?

National Comprehensive Cancer Network

"Survivorship" Clinical Practice Guidelines Version 1.2015

- Healthcare provider inquiries into treatment-related infertility should be made if indicated, with referrals as appropriate.

"Breast Cancer" Clinical Practice Guidelines Version 3.2015

- All premenopausal patients should be informed about the potential impact of chemotherapy on fertility and asked about their desire for future pregnancies.
- Those who may desire future pregnancy should be referred to a fertility specialist before chemotherapy.
- Patients should not become pregnant during treatment with radiation therapy, chemotherapy, or endocrine therapy.

American Society of Reproductive Medicine

"Fertility preservation and reproduction in patients facing gonadotoxic therapies: a committee opinion" (2013)

- At present, there do not appear to be major mutagenic effects in offspring born to patients successfully treated for cancer.
- The development of techniques for prenatal diagnosis and preimplantation genetic diagnosis provides a way that parents with heritable cancers can prevent transmission of that risk to offspring.
- If cancer survivors have retained reproductive function, they may conceive coitally. If they have diminished reproductive function, they may seek the help of fertility specialists. In some cases they can make use of previously stored gametes, embryos, and gonadal tissue for that purpose. Other options that may be appropriate include donor gametes, donor embryos, gestational surrogacy, and adoption.
- Apart from the risks posed by fertility treatment, physicians may be concerned about the risks posed by pregnancy on cancer recurrence. Reproductive physicians treating cancer survivors should be cognizant of the patient's medical status, treatment plan, and prognosis.
- Clinicians should inform patients receiving potentially gonadotoxic therapies about options for fertility preservation and future reproduction prior to the initiation of such treatment. A collaborative multidisciplinary team approach is encouraged.

American Congress of Obstetrics and Gynecology

"Management of gynecologic issues in women with breast cancer" (2012)

- Pregnancy after breast cancer is not thought to increase breast cancer recurrence.
- If future pregnancy is desired for women in whom breast cancer has been diagnosed, appropriate consultation with fertility specialists should be offered to ascertain whether immediate assisted reproductive strategies are possible to preserve fertility.

Children's Oncology Group Long Term Follow-up Guidelines

"Female reproductive health after childhood, adolescent, and young adult cancers: guidelines for the assessment and management of female reproductive complications." (2013)

- Healthcare provider inquiries on the following are recommended:
  - Annual pregnancy and childbirth history in survivors at risk of reduced fertility or adverse pregnancy outcomes
  - Menstrual cycle history, sexual function and libido
  - Laboratory screening including LH, FSH, and estradiol levels for all women who wish to become pregnant
- Survivors with normal ovarian function at risk of therapy associated ovarian failure should be counseled about this risk.
- Women unable to conceive should be referred to reproductive endocrinology.

American Society of Clinical Oncology

"Fertility Preservation in Patients with Cancer: American Society of Clinical Oncology Guideline Update" (2013)

- As part of education and informed consent before cancer therapy, healthcare providers should address the possibility of infertility with patients treated during their reproductive years and be prepared to discuss fertility preservation options and/or to refer all potential patients to reproductive specialists.

Fertility Concerns Resources

Geared toward survivors

What type of information is here?	What organization provides this resource?
Information on fertility options before and after treatment	American Cancer Society
Fertility preservation information for patients, parents, and partners	Oncofertility Consortium
Locate U.S. fertility specialists and view individual clinic IVF success rates	Society of Assisted Reproductive Technology
Navigation for cancer survivors on finding a reproductive endocrinologist and potential discounts on fertility services and medications, fertility risk calculator	LIVESTRONG Fertility
Forum for young breast cancer survivors to discuss fertility after cancer	Young Survival Coalition
Overview of adoption policies and link to finding an adoption attorney	American Academy of Adoption Attorneys
Audio recording discussing fertility, sexuality, and intimacy for young adult cancer survivors	Cancer*Care*

Geared toward healthcare providers

What type of information is here?	What organization provides this resource?
Information on fertility issues aimed at healthcare providers	National Cancer Institute
Locate U.S. fertility specialists and view individual clinic IVF success rates	Society of Assisted Reproductive Technology
Online training to help healthcare providers offer better fertility care to cancer patients	LIVESTRONG Fertility
Survivorship and Breast Cancer Clinical Practice Guidelines	National Comprehensive Cancer Network
Committee Opinion on fertility preservation in patients facing gonadotoxic therapies	American Society of Reproductive Medicine
Practice Bulletin on gynecologic issues in women with breast cancer	American Congress of Obstetrics and Gynecology
Guidelines on long term follow up of reproductive health	Children’s Oncology Group
Guidelines on fertility preservation before cancer treatment	American Society of Clinical Oncology
